# Neuronal Cell Adhesion Molecules May Mediate Neuroinflammation in Autism Spectrum Disorder

**DOI:** 10.3389/fpsyt.2022.842755

**Published:** 2022-04-15

**Authors:** Madeline Eve, Josan Gandawijaya, Liming Yang, Asami Oguro-Ando

**Affiliations:** University of Exeter Medical School, University of Exeter, Exeter, United Kingdom

**Keywords:** autism spectrum disorder, cell adhesion molecules, neuroinflammation, inflammatory cascade, neuroinflammatory signaling, glial cells

## Abstract

Autism spectrum disorder (ASD) is a complex neurodevelopmental condition characterized by restrictive and repetitive behaviors, alongside deficits in social interaction and communication. The etiology of ASD is largely unknown but is strongly linked to genetic variants in neuronal cell adhesion molecules (CAMs), cell-surface proteins that have important roles in neurodevelopment. A combination of environmental and genetic factors are believed to contribute to ASD pathogenesis. Inflammation in ASD has been identified as one of these factors, demonstrated through the presence of proinflammatory cytokines, maternal immune activation, and activation of glial cells in ASD brains. Glial cells are the main source of cytokines within the brain and, therefore, their activity is vital in mediating inflammation in the central nervous system. However, it is unclear whether the aforementioned neuronal CAMs are involved in modulating neuroimmune signaling or glial behavior. This review aims to address the largely unexplored role that neuronal CAMs may play in mediating inflammatory cascades that underpin neuroinflammation in ASD, primarily focusing on the Notch, nuclear factor-κB (NF-κB), and mitogen-activated protein kinase (MAPK) cascades. We will also evaluate the available evidence on how neuronal CAMs may influence glial activity associated with inflammation. This is important when considering the impact of environmental factors and inflammatory responses on ASD development. In particular, neural CAM1 (NCAM1) can regulate NF-κB transcription in neurons, directly altering proinflammatory signaling. Additionally, NCAM1 and contactin-1 appear to mediate astrocyte and oligodendrocyte precursor proliferation which can alter the neuroimmune response. Importantly, although this review highlights the limited information available, there is evidence of a neuronal CAM regulatory role in inflammatory signaling. This warrants further investigation into the role other neuronal CAM family members may have in mediating inflammatory cascades and would advance our understanding of how neuroinflammation can contribute to ASD pathology.

## Introduction

Autism spectrum disorder (ASD) is a complex neurodevelopmental condition characterized by restrictive and repetitive behaviors, combined with deficits in social interaction and communication (1). ASD is estimated to currently affect between 0.6 and 2% of the global population, with data suggesting prevalence is increasing over time (2–5). The etiology of ASD remains largely unknown, although it is hypothesized that a combination of environmental and genetic factors contributes to its pathogenesis (6). Monozygotic twin studies show ASD has high heritability, with estimates as great as 83%, but these figures also demonstrate the incomplete genetic concordance (7). ASD affects predominantly males compared to females for reasons yet unidentified (8).

Extensive, current research collated by the Simons Foundation Autism Research Initiative (SFARI) highlights numerous candidate genes linked to ASD development, with many of these encoding for neuronal cell adhesion molecules (CAMs) (9). Neuronal CAMs are together a diverse collection of cell-surface proteins that play roles in neurite formation, neuronal outgrowth, and axon guidance within the nervous system (10, 11). These molecules are classified into families founded on their structure, including the neurexin (NRXN) family, neuroligin (NLGN) family, and immunoglobulin superfamily CAMs (IgCAMs) (12). Amongst them, the IgCAMs represent the largest family of neuronal CAMs, encompassing neurofascins (NFASCs), neural CAMs (NCAMs), and the six-member subfamily, contactins (CNTNs) (12). Of note, variation in *CNTN* gene expression is linked to ASD, and various studies have demonstrated their importance in nervous system development (13). CNTNs have been reported to modulate neuronal communication and synaptic transmission, essential in the assembly of neural circuits and the formation of behavioral pathways (10, 12–15).

One key neuronal CAM, scored by the SFARI Gene database as a strong, syndromic ASD candidate gene is *CNTN-associated protein-2* (*CNTNAP2)* (16). CNTNAP2 is localized to myelinated axons within the juxtaparanode of the nodes of Ranvier. Here, it interacts with CNTN2 and organizes axonal voltage-gated K^+^ channels (17). Consistent evidence reports rare variants within *CNTNAP2* as a susceptibility factor for ASD and suggests that CNTNAP2 has a function in the early stages of neurodevelopment and later language development (18–21). Animal models deficient for Cntnap2 show symptoms synonymous with ASD in humans, including epileptic seizures, behavioral abnormalities, and cognitive dysfunction (22). Functional magnetic resonance imaging has illustrated atypical frontal cortex circuitry in children with ASD carrying common *CNTNAP2* variants, compared to neurotypical controls (21). A case report presented by Al-Murrani and colleagues depicted a 3-year-old boy with a deletion in *CNTNAP2* who displayed language delay, communication difficulties, and consequently, behavioral problems (23). However, it is important to note that two of his family members also carried the same deletion and yet exhibited no ASD phenotype. This demonstrates the presence of substantial phenotypic heterogeneity that makes it difficult to identify a solely genetic origin for many cases of ASD and, therefore, suggests an additional environmental trigger contributes to ASD symptom presentation.

Environmental factors can be categorized as prenatal, natal, and postnatal. Common prenatal risk factors include older paternal age, maternal mental and physical health, as well as familial socioeconomic status (24). A particularly promising avenue of research into the natal and postnatal environmental triggers for ASD is inflammation. The relationship between inflammatory disease, proinflammatory cytokines, and impaired immunity has been well-documented in those with ASD. Specifically, immune disorders such as asthma, gastrointestinal (GI) disorders, allergy, and maternal immune activation (MIA) have been regularly identified in studies investigating ASD comorbidities (25–30). MIA occurs when the pregnant mother acquires an infection or is exposed to immunogenic materials and has been identified to increase susceptibility to a child developing ASD (31). Consequences on the developing fetal brain can include modified expression of neuronal migration genes, increased number of microglia, aberrant dendritic morphology within the prefrontal cortex, and excessive neurogenesis (32–35). It is thought that the mother’s immune response poses a risk to fetal neurodevelopment, rather than the specific pathogen or immunogenic material (35). In murine models, ASD behaviors are observed in offspring when, throughout pregnancy, dams are exposed to immunostimulants such as lipopolysaccharide (LPS), interleukin-17 (IL-17), and polyinosinic-polycytidylic acid [poly (I:C)] (29, 36, 37). In one study, blocking IL-17A in LPS-immune activated dams resulted in the reversal of ASD behaviors in their offspring (36). These studies demonstrate that altered neuroimmune signaling adversely affects fetal neurodevelopment. Notably, MIA has also been shown to facilitate the transfer of maternal antibodies targeting fetal neural proteins, which could further impair neurodevelopment (38).

Although inflammation has been identified as an environmental risk factor for ASD, it is not entirely clear whether the neuronal CAMs discussed above may play roles in modulating neuroimmune signaling. Microglia are the main source of cytokines within the brain and, therefore, are vital in mediating inflammation in the central nervous system (CNS) (39). There is suggestion of neuronal CAMs influencing signaling cascades that then cause a proinflammatory phenotype in microglia (40).

**FIGURE 1:**
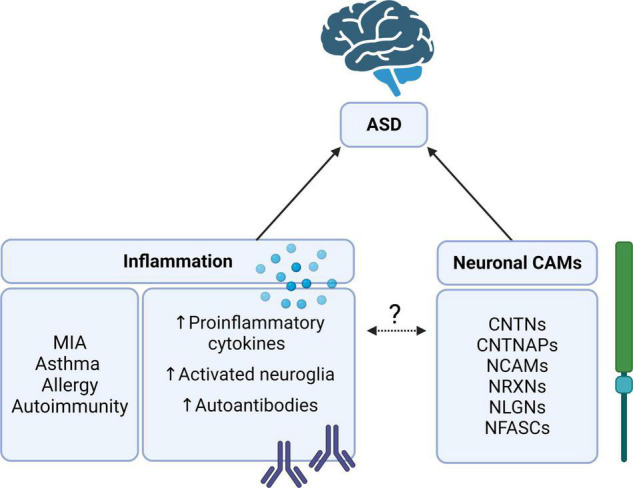
The relationship between autism spectrum disorder (ASD), inflammation, and cell adhesion molecules (CAMs). Neuronal CAMs such as the CNTN, CNTNAP, NCAM, NRXN, NLGN, and NFASC families of proteins are consistently implicated as risk genes for ASD (9, 16). Likewise, inflammation, particularly in MIA, has been identified as an environmental risk factor for ASD (24, 31). What is yet unclear is whether these neuronal CAMs may be involved in regulating inflammatory responses or cascades in ASD. Contactin (CNTN), CNTN-associated protein (CNTNAP), neural cell adhesion molecule (NCAM), neurexin (NRXN), neuroligin (NLGN), neurofascin (NFASC), and maternal immune activation (MIA). Created with BioRender.com.

Inflammatory processes from subsequent microglial activation indicate that neuronal CAMs may play a role in inflammatory cascades, a concept relatively unexplored in current literature. Studies of several CAMs, including CNTNAP2, have gradually revealed their functional involvement in inflammatory signaling. However, there is limited information that discusses the relation between CAMs and inflammatory systems that are risk factors in ASD. This review aims to address the association between neuronal CAMs and inflammatory systems within the scope of ASD (Figure 1). This association has been largely unexplored but is important when considering the impact of environmental factors and the inflammatory response on ASD. The following sections will cover recent research reports exploring the role of neuronal CAMs in inflammatory signaling cascades and proinflammatory cytokine production associated with neuroinflammation found in ASD. Additionally, we will focus on the dysfunction of inflammatory cascades underpinning inflammatory diseases that may link to ASD pathology. Finally, we will assess how neuronal CAMs are involved in the activation, differentiation, and proliferation of glial cells, leading to an inflammatory response and how this may impact neurodevelopmental pathways implicated in ASD.

## Autism Spectrum Disorder and Inflammation

### Signaling Pathways Involved in Neuroinflammation

Several signaling pathways are involved in the neuronal inflammatory response. This review will primarily focus on three of these; Notch, nuclear factor-κB (NF-κB), and mitogen-activated protein kinase (MAPK) signaling cascades, which all play key roles in inflammatory responses. Notch is a transmembrane protein that, once activated, transduces signals *via* direct cell-cell communication (41). There are four Notch receptors in mammals that, upon binding with a corresponding ligand, causes the intracellular domain of the Notch receptor to be cleaved and translocated to the nucleus (42). Importantly, neuronal CAMs, such as CNTN1 and CNTN6, act as ligands for Notch1 in the CNS (43, 44). In the immune system, Notch1 signaling is important for the differentiation of T cells into T-helper (Th) and regulatory T cells, whilst inhibiting the differentiation of other lymphoid lineages (45–47). Chronic inflammatory diseases associated with ASD, such as inflammatory bowel disease (IBD) and asthma, may be influenced by Notch signaling (41). Experimental alterations of the Notch signaling pathways, such as the removal of constituents responsible for Notch signal transduction, have exposed a potential role of Notch in the proinflammatory response (48, 49). Moreover, proinflammatory cytokine IL-6 is secreted upon activation of Notch2 by tumor necrosis factor-α (TNF-α) in rheumatoid arthritis models (50). Hence, inhibition of Notch signaling may be beneficial in the treatment of inflammatory diseases. However, whether neuronal CAMs acting as Notch ligands modulate the secretion of proinflammatory cytokines is unclear.

NF-κB is another signaling pathway involved in inflammation and is strongly implicated in inflammatory disease. Present in all cell types, NF-κB regulates the expression of genes encoding for proteins vital in the immune response (51). Activation of NF-κB is inducible by inflammatory cytokines, markers of infection, or stress-activated protein kinases (52). Under normal cell conditions, NF-κB is inhibited from nuclear translocation by IκB (inhibitor of κB) until activated (53). NF-κB signaling influences differentiation states of cells and regulates the production of anti-apoptotic factors (54). However, the primary role of NF-κB is to increase the production of cytokines, chemokines, and adhesion molecules involved in the immune response (55). Proinflammatory cytokines such as IL-6, IL-12, and TNF-α form common target genes for activated NF-κB (56). Although intentional activation of NF-κB is protective against pathogens and cancer, uncontrolled or dysfunctional NF-κB signaling can be causative of acute or chronic inflammatory disease (52). Within the CNS, inappropriate activation of the NF-κB signaling cascade leads to aberrant expression of proinflammatory cytokines, which can be particularly damaging. NF-κB activation in microglia, astrocytes, and oligodendrocytes has been implicated in neurodegenerative disease (57). Of note, NF-κB signaling can also be initiated downstream of the MAPK cascade (58).

The MAPK signaling cascade is activated by cytokines, mediators of stress, and inflammatory markers, such as ligands of Toll-like receptors (59). Through a series of phosphorylation cascades, MAPK proteins transduce, and propagate extracellular signals within the cell (60). During inflammation, MAPK signaling is mainly induced by Toll-like receptor activation, resulting in the phosphorylation of transcription factors (61). Once phosphorylated, these transcription factors translocate to the nucleus and transcribe genes encoding proinflammatory cytokines, such as IL-6 and TNF-α (62). Within the CNS, IL-6 signaling forms a positive feedback loop through activation of MAPK signaling, which is reported to promote neurogenesis (63). Microglial activation can also induce the MAPK signaling cascade, promoting a neuroinflammatory environment that contributes to neurodegeneration (64). Although it is uncertain, neuronal CAMs may also play roles in MAPK signaling. For example, NCAM1 was shown to activate MAPK signaling in mesenchymal stromal cells, regulating their migration to the site of inflammation where they contribute to tissue repair (65).

During an inflammatory response, Notch, NF-κB, and MAPK signaling pathways can all be activated by proinflammatory cytokines (52, 59, 66).

### Cytokine and Chemokine Profiles in Autism Spectrum Disorder

In the context of the immune system, cytokines are proteins secreted by cells to coordinate, signal and recruit, normally in response to an immune insult. Generally, these signaling proteins are proinflammatory in nature and are important in orchestrating the immune response (67). Most cell types can produce cytokines, but the majority of proinflammatory cytokines are released by macrophages and lymphocytes (68). Chemokines, for example, IL-8, manage leukocyte adhesion and chemotaxis (69). Interleukins play a role in directing cell differentiation, growth, and activation (68, 70). IL-1 and IL-6 are, for the most part, produced by macrophages to stimulate the generation of proinflammatory cytokines and evoke elevation of body temperature (70). The growth and proliferation of eosinophils, T cells, natural killer cells, and B cells are mediated by a vast range of interleukins (70). Cytokines can also be anti-inflammatory and regulate or suppress an escalating immune response. For instance, IL-10 suppresses proinflammatory cytokines and modulates macrophages to dampen the immune response (71, 72).

Irregular cytokine and chemokine profiles have been recorded in the cerebrospinal fluid (CSF), blood, and brain of those with ASD (73–76). Plasma samples from male ASD subjects revealed elevated levels of IL-1β, IL-5, IL-8, IL-12, IL-13, and IL-17 compared to controls (76). In another multiplex cytokine screen, similar differences were found in IL-1β, IL-12, and IL-17, but also IL-6, in individuals with ASD compared with age-matched typically developing children (77). A recent meta-analysis conducted with 1,393 patients with ASD found greater concentrations of IL-6 in the blood, as well as increased concentrations of proinflammatory cytokines IL-1β, TNF-α, and interferon-γ (IFN-γ) within the periphery (78). Within the CNS, localized cytokine and chemokine variances have been reported. Higher levels of IL-6, IL-8, TNF-α, and IFN-γ are observed in the frontal cerebral cortex of ASD patients compared to age-matched control cortices (79). Moreover, evidence of elevated TNF receptor I levels in the CSF of ASD children further indicates inflammation not just in the periphery, but also within the CNS (80). Although inflammatory markers may differ depending on genetic predisposition, when screening panel results are normalized to parental cytokine expression, IL-8 expression was still seen to be elevated in children with ASD (81). These cytokine profiles have been found to differ in males and females, indicating that testosterone may impinge on the inflammatory processes underlying ASD (82). Cytokine profiles may also be useful as biomarkers to predict comorbidities. Children with ASD that also suffered from epilepsy had lower peripheral IL-6 levels compared to ASD children without epilepsy (77). Distinct cytokine profiles have also been shown in ASD children who have attention deficit hyperactivity disorder, with differences in blood IL-8 levels compared to children with only ASD (74). Specific cytokine profiles have the potential to act as an ASD biomarker and aid the diagnosis of comorbidities.

For a more detailed overview of which cytokines and chemokines are most frequently found to vary in concentrations within individuals with ASD compared to typically developing controls, see Table 1. In summary, IL-1β, IL-6, IL-8, and IL-17 are the most consistently observed cytokines to be upregulated in cases of ASD, which indicates a proinflammatory response underlying neuroinflammation in ASD.

**TABLE 1:** Summary of cytokines and chemokines reported to have altered expression in autism spectrum disorder.

Cytokine or chemokine associated with ASD	References
IL-1	(76, 78, 88, 90, 184)
IL-2	(88)
IL-4	(88, 109)
IL-5	(76)
IL-6	(73, 75, 78, 79, 88, 90, 100, 116, 118, 184, 256)
IL-7	(81, 88)
IL-8	(76, 79, 81, 88, 90)
IL-10	(114)
IL-12	(76, 88, 90)
IL-13	(76)
IL-17	(26, 29, 73, 75, 76, 88, 114, 116, 117, 184)
IL-23	(257)
TNF-α	(73, 78, 79, 88, 184)
IFN-α	(81)
IFN-γ	(78, 79, 81, 109)

*ASD, autism spectrum disorder; IL, interleukin; TNF, tumor necrosis factor; IFN, interferon.*

### Interleukin-1β

Interleukin-1β is a proinflammatory cytokine found upregulated in autoinflammatory disease, chronic inflammation, and acute inflammation (83). Expression of IL-1β by monocytes and macrophages, stimulated by pathogen-associated molecular patterns and cytokines, triggers phagocytic cell activation (84). IL-1β has a role in neuropathogenesis but also neuroprotection within the CNS (85). During the development of the nervous system, IL-1β expression regulates the proliferation of neural progenitor cells (86). Hence, abnormal IL-1β levels may contribute to neurological deficits observed in ASD brains. Increased concentrations of IL-1β were shown in several studies, including a meta-analysis, in individuals with ASD compared to healthy controls (76, 78, 87, 88). Microglia can generate vast quantities of cytokines, particularly IL-1β, within the CNS (89). Extracellular vesicles isolated from the serum of children with ASD were shown, *in vitro*, to activate microglia to produce increased levels of IL-1β compared to neurotypical controls (40). Interestingly, IL-1β is found to be elevated in the serum and blood of both males and females with ASD, although mainly males have been included in ASD cytokine profile studies (76, 82). This increase is not as high of an increase as would be expected to be found in a person with autoimmune or inflammatory disease, suggesting that the effect of ASD on the inflammatory system is unique (76). One study showed that increased IL-1β was predominantly found in children with regressive ASD, supporting that IL-1β is influential during post-natal neurodevelopment (90). The same study also found a correlation between IL-1β concentration and aberrant behaviors (90).

### Interleukin-8

Interleukin-8 production in neutrophils and mast cells is stimulated by IL-1β (91). IL-8, also known as CXCL8, is a potent chemoattractant produced by T cells and macrophages in order to recruit neutrophils and other leukocytes to a site of inflammation (92). Neutrophils can produce IL-8 to self-recruit, stimulated by IL-1 and TNF-α (92). Besides neutrophil chemotaxis, IL-8 has a role in neutrophil morphology, upregulation of adhesion molecules, migration, and exocytosis of proteolytic enzymes (92, 93). Although crucial in peripheral immunity, IL-8 has also been found to be increased within the frontal cerebral cortex of ASD brains (79). The frontal cortex is essential for cognition, emotion, and social behavior, therefore, is a brain region associated with the pathology of ASD (94). Localized inflammation due to IL-8 could affect frontal cortex processing in those with ASD. IL-8 is produced by macrophages to recruit neutrophils, eosinophils, and leukocytes (95). Increased peripheral levels of IL-8 have been found in ASD subjects compared to matched controls (76, 87, 88, 96). With IL-8 having such a key role in innate immunity, it suggests that immune dysfunction in ASD is linked to the innate immune system. Activated microglia may be able to recruit cells of the innate immune system *via* IL-8 secretion to exacerbate the neuroinflammatory response. One study showed that not only was serum IL-8 concentration increased in ASD children compared to healthy controls, but IL-8 was yet higher in concentration in patients with childhood ASD compared to those with Asperger syndrome (96). This data may allude to different pathophysiological mechanisms for different levels on the ASD spectrum. Once more, an increase in severity of ASD phenotype is correlated with proinflammatory cytokine concentration (90). IL-6 plasma concentrations are also demonstrated to be positively correlated with the severity of ASD traits (90).

### Interleukin-6

Interleukin-6 is a pleiotropic cytokine that has both inflammatory and anti-inflammatory purposes throughout the human body (97). Functions of IL-6 during inflammation include B-cell differentiation, induction of acute-phase protein release, inhibition of regulatory T-cell differentiation, and maintenance of Th17 cell differentiation (70, 97). IL-6 activity is not limited to the immune system. It is well-documented to play roles in neurodevelopment, such as promoting neurite outgrowth, neurogenesis, and gliogenesis pathways that are implicated in ASD pathology (6, 94, 98–100). Many studies have shown increased plasma and blood IL-6 levels in individuals with ASD (73, 75, 76, 78, 88). Elevated IL-6 levels within the cerebellum are associated with impairment of neuronal cell adhesion and migration, as well as influencing synapse formation (101). CAMs are essential for the formation of behavioral pathways, synapse development, and neuronal plasticity, therefore, CAM dysfunction due to IL-6 may contribute to some ASD pathophysiologies (12, 13, 101). What is more, this data suggests IL-6 may regulate the function of neuronal CAMs (101).

There are distinguished neurological changes in the brains of those with ASD such as neuronal overgrowth in the frontal cortex and microglial activation (94, 102). In the GFAP-IL-6 mouse model of chronic neuroinflammation, where IL-6 is overexpressed in astrocytes, neurological variations like astrocytic gliosis and neurodegeneration were observed (103, 104). Increases in IL-6 serum, blood, and CSF levels have also been observed in adults with ASD, indicating IL-6 can be upregulated over a prolonged period of time, potentially contributing to neurological changes in ASD brains, similar to those observed in chronic neuroinflammation models (78, 102).

An increase of IL-6 was found in the anterior cingulate gyrus of ASD patients compared to controls (102). The anterior cingulate gyrus is responsible for emotional expression, attention allocation, and mood, the dysregulation of which are all core ASD deficits (105). Localized inflammation within this brain region *via* IL-6 could contribute to deficits in communication observed in those with ASD.

IL-6 appears to have a key role in MIA, one of the strongest examples of inflammation associated with ASD development. The immune response of the mother, directed against a pathogen or immunogenic molecule, adversely affects fetal neurodevelopment (31). In animal models, MIA is commonly induced in pregnant mice *via* administration of LPS or poly(I:C), which mimics bacterial or viral infection, respectively (106). Behavioral changes in the offspring of poly(I:C)- and LPS-induced MIA mice were observed in multiple studies (29, 36, 37, 107–109). These behaviors included enhanced marble-burying or self-grooming (represents repetitive and restricted behavior), sociability impairments, and reduced ultrasonic vocalization (which may suggest a change in social communication) (36, 108). Administration of IL-6 to pregnant dams gave rise to ASD-like behavioral traits in their offspring and further experiments utilizing poly(I:C) MIA models found offspring ASD behavior was prevented by co-administering anti-IL-6 antibodies with poly(I:C) (110). In this same study, MIA IL-6 knockout mice sired offspring without any of the behavioral deficits that MIA wild-type offspring possessed (110). Together with the upregulated IL-6 levels observed in human studies, there is substantial evidence implicating altered IL-6 signaling in the neuroimmune dysfunction underlying ASD.

### Interleukin-17

Interleukin-17 is an important cytokine in the protection and clearance of bacterial and fungal infections (111). There are six members of the IL-17 family, the most studied of which is IL-17A (112). Able to act on myeloid and mesenchymal cells, IL-17 upregulates proinflammatory genes through NF-κB and MAPK signaling (113).

Th17 cells are characterized by their production of IL-17 and it has been suggested that Th17 cells are implicated in the development of inappropriate inflammatory responses in ASD (114, 115). A study conducted by Moaaz *et al.* found children with ASD had significantly increased Th17 cell production, alongside fewer regulatory T cells and decreased concentrations of both IL-10 and transforming growth factor-β (TGF-β), which dampen the inflammatory responses (114). This could indicate that regulation of the immune system may be malfunctioning in some cases of ASD.

Increased plasma levels of IL-17 in those with ASD have been outlined in numerous studies (73, 77, 114, 116). Additionally, increased expression of the receptor for IL-17A was found in phagocytes isolated from individuals with ASD, and IL-17 messenger RNA expression was also nearly four times higher in ASD children compared to typically developing children (114, 117, 118). One Turkish study detected reduced IL-17 expression in ASD individuals’ peripheral blood mononuclear cells (PBMC), though this could be due to demographic differences within the sample (75). The proinflammatory IL-17 signaling pathway is associated with chronic inflammatory neurological diseases (119). Activation of IL-17 receptors (IL-17R) *in vitro* in monocytes and neutrophils isolated from individuals with ASD led to upregulated NF-κB expression, resulting in increased expression of proinflammatory genes (117, 118). Application of anti-IL-17R antibody to ASD monocytes reversed this enhanced NF-κB expression and could be viewed as a beneficial treatment for managing the inflammation found in some cases of ASD (117).

The above data suggests that upregulation of IL-17 is associated with ASD, but the origin of IL-17 dysregulation may be maternal. In pregnant mothers, IL-17 is able to transfer from the placenta to the fetus, increasing IL-17R levels within the fetal brain (120). Additional IL-17R further increases IL-17 signaling within the fetal brain, likely initiating a neuroinflammatory response (121). This may cause a predisposed sensitivity to IL-17 and explain increased IL-17 concentrations in individuals with ASD. Abnormal IL-17 levels have featured in MIA models. IL-6 and TGF-β together facilitate Th17 cell differentiation from their CD4^+^ progenitors (115). Th17 cells are characterized by their production of IL-17. A rise in Th17 cell numbers is associated with autoimmune disorders and chronic inflammatory disease (122). It has been found that an increase in serum IL-6, ensuing from an immune assault, yields an elevated production of Th17 cells, increasing IL-17 levels (115). Interestingly, in two studies, the enhanced marble-burying behavior was ameliorated by IL-17A blocking, indicating that IL-17A has a role in repetitive ASD behavioral traits (36, 107). Blocking IL-17A in LPS-administered MIA dams also reversed the reduction in ultrasonic vocalization and social interaction deficits in their offspring (36). Increased levels of maternal IL-17A have been strongly associated with ASD-like behavior in rodent MIA offspring. This amplification of IL-17 has additionally been noticed in both murine MIA offspring and human ASD individuals.

In summary, specific cytokine profiles seem to highlight the presence of a proinflammatory response in individuals with ASD during early neurodevelopment, up to adulthood. Attention is drawn to abnormally elevated IL-1β, IL-6, IL-8, and IL-17 levels in children and adults with ASD (73, 74, 96). Increased IL-6 and IL-17 levels in MIA models suggest that dysregulation of inflammatory cascades begin during prenatal neurodevelopment (100). There is also some evidence that the degree of severity in ASD phenotype may be positively correlated with the concentrations of proinflammatory cytokines (90). Activation of inflammatory signaling in glial and neuronal cells by proinflammatory cytokines may alter neuronal cellular function. Significantly, IL-6 overexpression can promote astrocytic gliosis, causing neuroinflammation similar to that observed in ASD brains (104). In turn, the neuroinflammatory environment activates MAPK and NFκB signaling to enhance the neuroimmune response (52, 59). These inflammatory cascades can be regulated by NCAM1, implicating neuronal CAMs in ASD-related inflammation (65, 123). Additionally, extracellular vesicles isolated from ASD serum may activate microglia to produce increased IL-1β, further suggesting dysfunction of the neuroinflammatory response (40). IL-1β regulates the proliferation of neural progenitor cells, therefore, an increase in IL-1β in the brains of those with ASD may alter neuronal development (86). This may have implications in ASD behavioral pathways that contribute to the core deficits seen in ASD.

## Involvement of Neuronal Cell Adhesion Molecules in Inflammatory and Immune Disease Associated With Autism Spectrum Disorder

### Inflammatory and Immune Disease Associated With Autism Spectrum Disorder

Dysregulation of inflammatory signaling cascades and abnormal proinflammatory cytokine signaling is often present in those with ASD (77). Chronic inflammatory diseases such as asthma, IBD, and persistent neuroinflammation have frequently been reported as comorbidities to ASD, alongside immune-mediated disease (124).

Asthma is a chronic inflammatory disease of the respiratory system (125). Current literature is divided over support of a correlation between asthma and ASD. A meta-analysis of 175,406 participants found no proof of an association between asthma and ASD (126). Another study also showed no association between ASD and asthma or allergy, although allergy was linked with increased repetitive behavior (127). The same study did, however, highlight that food allergies and sensitivities were associated with ASD. Other studies report that asthma is 35% more frequently diagnosed and is more prevalent in children with ASD than in typically developing controls (26, 128). Interestingly, PBMCs isolated from children with both ASD and asthma are reported to produce higher levels of IL-17 following stimulation compared to PBMCs from children with ASD but without asthma (26). It is worth noting that upregulated IL-17, as previously discussed (previous Interleukin-17 section), can contribute to more severe ASD phenotypes.

Gastrointestinal sensitivities and general GI issues are further comorbidities to ASD (124). Children with ASD are frequently reported to have intolerances to food, abdominal pain, bloating, diarrhea, constipation, ulcerative colitis, and Crohn’s disease (129). There has been a lot of interest in the “gut-brain axis,” where gut microbiota and immune responses have a bidirectional relationship with the CNS (130). Children aged 2–18 with ASD had 67% higher odds of having Crohn’s disease and ulcerative colitis compared to a typically developing control group (131). Across four different study populations, the rates of IBD among individuals with ASD were higher than their age-matched controls (132). Impaired gut barriers from localized inflammatory cytokine production leads to increased gut permeability, allowing cytokines to access the CNS (133). These cytokines, originating from the gut, could instigate inflammatory responses within the brain and, therefore, impact cognitive function (130). Reciprocally, inflammation originating from the CNS or plasma could cross the intestinal mucosal barrier and trigger inflammatory signaling in gut-associated lymphoid tissue (133, 134).

The gut-brain axis may indirectly play a role in the neuroimmune system. Increased gut permeability from GI inflammation allows molecules that would otherwise be restricted from entering the bloodstream (30). Microglia are primarily responsible for neuroinflammation within the CNS and, once activated, produce vast quantities of proinflammatory cytokines (89). Activation of microglia and astrocytes were shown in brain tissue of patients with ASD (102). The vagus nerve interacts with the peripheral immune system, constantly surveying gut health (135). GI inflammatory markers can be sensed by the vagus nerve which transmits this information to the CNS, affecting microglial activation (136). Therefore, inflammation from the gut can trigger proinflammatory cytokine secretion *via* microglia activation, causing neuroinflammation (135).

Similar to ASD, autoimmune disease is thought to develop through a genetic predisposition with an environmental trigger that activates the immune system (137). The connection between autoinflammatory disease and ASD is not well-defined amongst current literature. However, several previous studies have revealed an interesting relationship between ASD and autoimmune disease. Zerbo *et al.* discovered autoimmune disease and psoriasis were diagnosed more frequently in males and children over the age of 12 with ASD compared to controls (138). It has also been established that a familial history of autoimmune disease may increase the chance of offspring having ASD, especially when the disease is targeting the CNS, and skin or mucosal membranes (139). These results suggest that there are overlaps in the genetic predisposition to ASD and autoimmune disorders (particularly those affecting the CNS). Interestingly, an autoimmune disease in the pregnant mother of ASD children may predispose the child to an IL-17 sensitivity *via* MIA and as described previously, an increase in IL-17 and the Th17 cells that produce it, is linked to the development of autoimmune disorders (119, 120).

Autoantibodies against proteins found within the CNS of subjects with ASD, including myelin basic protein, have been reported in several studies (140–142). Myelin is important in nerve function and protects the axon from damage. Without myelin, nerves cannot effectively conduct electrical signals in the CNS, resulting in neuronal network dysfunction (143). Demyelination, resulting from inflammation and cytokine infiltration, is a leading cause of neurological disease, impacting sensory, motor, and cognitive function (144, 145). White matter denotes brain regions consisting mainly of myelinated axons (145). Alterations in white matter volume of ASD brains, detected through magnetic resonance imaging, suggests dysregulation of myelination in those with ASD (146). It must be acknowledged that although demyelination is not commonly reported in those with ASD, the presence of autoantibodies against myelin basic protein in those with ASD suggests the potential for demyelinating disease to affect ASD brains (140). Additionally, one case study did present a 6-year-old with ASD and demyelinating neuropathy (147).

Evidence reports that neurodegenerative disease is more prevalent in adults with ASD (148). Alzheimer’s disease (AD) is a neurodegenerative disease that has similar mechanisms supporting the pathogenesis of ASD (149). A key protein implicated in the pathology of AD is amyloid precursor protein (APP). Aberrant cleavage of APP into toxic amyloid beta (Aβ) plaques are one of the hallmarks of AD, although several studies reveal similar alterations of APP processing in those with ASD (150). A significant increase in secreted β-amyloid, a product of the pathogenic APP processing pathway, have been found in the plasma of children with severe ASD (151). Additionally, a greater intraneuronal Aβ load and increased Aβ accumulation were observed in astrocytes and some microglia in subjects with ASD and 15q11.2-13q duplication syndrome (152). Activated microglia and astrocytes clear Aβ plaques, reducing their accumulation at the synapse (153, 154). In AD models, microglia respond more readily to Aβ, producing increased proinflammatory cytokines that degrade neuronal synapses, leading to cognitive decline (155). Moreover, transgenic AD mice models showed that increased expression of *APP* led to elevated levels of proinflammatory cytokines, such as IL-1β and IFN-γ, in the brain (154). An increased Aβ load, as demonstrated in those with ASD, would suggest enhanced astrocyte and microglial activation, similarly, increasing proinflammatory cytokine production (152). Furthermore, in the ASD brain, astrocytes and microglia may respond more readily to Aβ, damaging synapses that could contribute to altered cognition (155).

To review, there is conflicting data for the association of asthma with ASD, but there is a strong correlation between allergy and ASD (126). The GI system is particularly susceptible to environmental triggers through ingested materials. Individuals with ASD may have a predisposition to GI disorders that cause an inflammatory immune response once activated by environmental factors. Inflammation originating from the gut may be influencing inflammatory signaling in the CNS attributable to the gut-brain axis (135). Alternatively, GI inflammation may be instigated by inflammatory signaling deriving from the CNS. There is some evidence to link autoimmunity and dysregulation of immune function to ASD, although there is a lack of support for the presence of autoantibodies in those with ASD (140). More so, there is a suggestion that myelin dysregulation may render axons vulnerable to proinflammatory cytokines that cause demyelination and cognitive impairment (144). Finally, ASD has a complex pathogenesis involving a neuroinflammatory environment comparable to some aspects found in AD.

### Contactins and Contactin-Associated Proteins in Inflammatory and Immune Disease

Alongside a documented link between ASD and inflammatory disease, current literature (Table 2) also supports an association between CNTNs and CNTNAPs with inflammatory and immune-mediated disease.

**TABLE 2:** Overview of inflammatory diseases and immune disorders that are associated with neuronal cell adhesion molecules.

Inflammatory disease	Associated gene	References
Asthma and allergy	*CNTN1*	(156)
	*NRXN1*	(193, 194)
Gastrointestinal dysfunction	*NLGN3*	(207, 210–212)
Chronic inflammatory demyelinating polyneuropathy	*CNTN1*	(158, 160–165, 167–174, 176–178)
	*CNTNAP1*	(158, 162, 163, 167, 173, 174)
	*CNTNAP2*	(169)
	*NFASC*	(160–166, 168, 170, 175)
Multiple sclerosis	*CNTN2*	(180, 181)
Neurodegenerative disease	*CNTN2*	(190)
	*CNTN4*	(192)
	*NRXN3*	(201, 204)

*CNTN, contactin; CNTNAP, CNTN-associated protein; NRXN, neurexin; NLGN, neuroligin; NFASC, neurofascin.*

Recent exosome research has identified a link between CNTN1 and asthma. CNTN1 was found to induce Notch2 signaling in asthma to activate Th17 and Th2 cells (156). CNTN1 is present on the surface of exosomes containing allergens and acts as a Notch2 ligand for monocyte-derived dendritic cells (156). These dendritic cells secrete IL-4, IL-5, IL-6, IL-13, and IL-17A to drive an enhanced inflammatory response in the airways, suggesting that CNTN1 may act as an inflammatory mediator in the pathology of asthma (156).

Likewise, chronic inflammatory demyelinating polyneuropathy (CIDP) has been strongly correlated with CNTNs alongside additional associations to CNTNAPs. CIDP is an immune-mediated neuropathy, caused by damage to the myelin sheath, with large heterogeneity (157). Antibodies against neuronal CAMs such as CNTN1, CNTNAP1, CNTNAP2, and NFASC have been found in the periphery of patients with CIDP (158–175). All these CAMs are localized to the nodes and paranodes of myelinated axons within the CNS (159). The most common autoantibody shown in seropositive CIDP patients were anti-CNTN1 IgG4 subclass antibodies (169, 176). Cytotoxic effects on cerebellar neurons were identified with chronic administration of IgG4 anti-CNTN1 serum of a patient with CIDP (177). Proteins in the CSF of seropositive CIDP patients show probable blood-brain barrier breakdown, which could increase the likelihood of anti-CNTN1 antibodies entering the brain parenchyma (163, 178). It was demonstrated that CNTN1 expression was reduced in dorsal root ganglion neurons and cerebellar granule neurons after long-term exposure to anti-CNTN1 autoantibodies (177). Notably, Vargas *et al.*, showed that microglia and astrocytes within the cerebellum of ASD patients were activated upon the degeneration of granule cells within their vicinity (102). This raises the possibility that anti-CNTN1 antibodies targeting the cerebellar granule cells cause degeneration resulting in the activation of microglia and astrocytes. These then go on to produce proinflammatory cytokines. Like ASD, within the CSF of CIDP patients, high concentrations of proinflammatory cytokines have been observed (179). Interestingly, anti-CNTNAP2 IgG4 and anti-CNTN1 IgG1 antibodies were unable to cross the paranodal barrier, indicating these autoantibody subtypes may be less pathogenic than anti-CNTN1 IgG4 (169). However, once anti-CNTN1 IgG4 antibodies progressively deteriorate the paranode, other CAM autoantibodies may then be able to pass the paranodal barrier and accelerate demyelination.

Similar antibodies against CNTNs have been found in some cases of multiple sclerosis (MS) (180). Anti-CNTN2 antibodies were reported in a patient with MS, alongside CNTN2-specific T cells. These CNTN2-specific T cells were able to cause permeations in the blood-brain barrier and were revealed to form cortical lesions in animal models (181). Cortical lesions are the result of the inflammatory response against the myelinated sheath and can cause cognitive impairment (182). Although this data may not be directly applicable to ASD, MS demyelination pathology may provide insight into pathogenic mechanisms in ASD cases where CNS antibodies are present (140).

It has been established that the occurrence of neurodegenerative diseases is greater in adults with ASD (148). Evidence of chronic inflammation, such as activated microglia and proinflammatory cytokines, have been shown in the brains of patients with ASD, similar to that of AD (183, 184). The Notch signaling pathway regulates neurogenesis, axon guidance, and synaptic plasticity but in most cell types, can also initiate proinflammatory signaling cascades (41, 185). Therefore, dysfunction of the Notch signaling pathway has been thought to have implications in AD pathophysiology (185). By interacting with Notch1, CNTN1 may influence downstream inflammatory effects such as the expression of proinflammatory cytokines (e.g., IL-6 and IL-17) (41, 43, 186, 187). Notch signaling is found ubiquitously within the human body and is responsible for the homeostasis of many functions and for that reason, is hard to target pharmaceutically (41). However, anti-inflammatory therapies aimed at blocking Notch signaling may be able to be CNS-targeted through specifically impeding CNTN1-driven Notch signaling (188). This could be a useful approach to target neuroinflammation in ASD brains arising from aberrant Notch signaling.

As illustrated previously, Aβ has a key role in the neuropathology of AD (189). Interestingly, the CNTN family of proteins may play roles in regulating this process. Lower CNTN2 expression is observed in and around Aβ plaques that were within the hippocampus of patients with AD compared to controls (190). Additionally, CNTN2 concentrations within the CSF of those with AD were significantly reduced (190, 191). Although it is unclear what the relationship between CNTN2 and APP is, this may point toward a role for CNTN2 in the modulation of Aβ production. A similar role has been posited for CNTN4, which is known to interact with APP and promote its processing *via* the non-amyloidogenic (non-pathogenic) pathway (192). Altered expression of CNTN2 and CNTN4 in ASD could result in increased amyloidogenic processing of APP to Aβ, leading to plaque formation and subsequent inflammation. Furthermore, considering that CNTN2 expression in AD is correlated with increased expression of IL-1β and IFN-γ, it could be pertinent to explore how the CNTN proteins may facilitate Aβ production in ASD.

### Neurexins in Inflammatory and Immune Disease

Neurexin 1 has also been implicated in asthma and ASD pathology (Table 2). In one study, 43% of patients with a 2p16.3 deletion in NRXN1 were reported to have ASD and of these, 33.5% suffered from asthma and/or allergies (193). Similarly, a *de novo* mutation in NRXN1α was found in a child exhibiting ASD-associated behaviors and developmental delay, alongside asthma that required recurrent hospitalization (194). NRXN1 is co-expressed with CNTN1, suggesting they may be under control of similar transcriptional regulatory programs and have similar regulatory roles in asthma to CNTN1 (195–198). Nonetheless, there is currently no evidence to support a direct link between NRXN1 and asthma, likely indicating asthma occurrence in these case studies may be unrelated to NRXN1 mutations.

In addition to the CNTNs, NRXNs may also play a role in Aβ-induced neuroinflammation. NRXNs interact with Aβ oligomers that are located between deteriorated synapses, as found in pathogenic AD brains (200, 199). NRXN3 is expressed within the hippocampus and cerebral cortex, two important regions of the brain for memory and cognition (201, 202). Variants of the *NRXN3* gene have a strong association with ASD, but not much is known about its function within the scope of AD (16, 203). It was discovered that expression of NRXN3 was reduced in the hippocampus of those with AD and that this expression was inversely correlated with NLRP3 (NOD-, LRR-, and pyrin domain-containing protein 3) expression, which is a constituent of the inflammasome (204). NLRP3 inflammasome signaling leads to the production of IL-1β and IL-18, upon activation by a pathogen or cellular damage (205). This dysregulation of NRXN3 may allow deterioration of neural synapses, causing cellular damage (200). Cellular damage could activate NLRP3, resulting in the release of proinflammatory cytokines, instigating AD pathogenesis (205). Similar disruption in the brains of those with ASD could occur, equally triggering the NLRP3 inflammasome secretion of proinflammatory cytokines IL-1β and IL-18, contributing to the neuroinflammatory environment.

### Neuroligins in Inflammatory and Immune Disease

Available data indicates that NLGNs play a role in GI inflammation (Table 2). The ADAMs (a disintegrin and metalloproteinase) family are enzymes that are able to cleave transmembrane neuronal CAMs including NLGNs and NRXNs, both of which are associated with ASD (16, 206). ADAMs are expressed throughout the human body but notably, ADAM10 and ADAM17 are found both in the CNS and intestines (207). ADAM17 regulates GI and neural inflammation through the cleavage of TNF-α (increased TNF-α cleavage promotes inflammation) (208). Genetic studies have revealed that whilst ADAM17 expression decreases with age in control groups, in individuals with ASD, *ADAM17* expression increases with age (209). Increased ADAM17 expression in those with ASD facilitates increased TNF-α-mediated inflammation in the gut and brain. Moreover, increased cleavage of NLGN3 by ADAM10 may be causative in decreased intestinal transit seen in people with ASD. Nlgn3-deficient mice were shown to have increased colonic motility, suggesting impaired control of gut motility by the enteric nervous system (210). Like ADAM10 and ADAM17, *NLGN3* is expressed in both the GI system and the CNS (16, 207). *Nlgn3* mutant mice, which display ASD-associated behaviors, were found to have GI symptoms affecting the small intestine and colon function (211). Cecal weight was also decreased in Nlgn3-deficient ASD mice models, alongside increased density of enteric macrophages (212). Due to NLGN3’s role in both CNS and enteric systems, mutations affecting this gene have apparent consequences to the immune system.

### Neural Cell Adhesion Molecules in Inflammatory and Immune Disease

Despite there being little evidence to support the involvement of NCAM in inflammatory and immune disease, there is some indication of NCAM1 mediating inflammatory cascades that underlie inflammatory disease. Distinct gene expression profiles were found within GI mucosal tissue in people with ASD and GI problems. However, these profiles overlapped significantly with transcriptome profiles of those with IBD, proposing a unique ASD-associated IBD variant. Genes that were exclusively differentially upregulated in ileal and colon samples from the ASD-GI group, compared to neurotypical IBD patients, included *IL-2 receptor alpha (IL2RA)* and *IL-4-induced 1 (IL4I1)* (213). IL4I1 promotes CNS remyelination and IL2RA can activate the MAPK signaling pathway (214, 215). Both remyelination processes and MAPK signaling have been associated with NCAM1 (65, 216). Not only does this link ASD with inflammatory GI disorders, but it may also implicate neuronal CAMs in their pathophysiology’s. Due to the ambiguous nature of this potential relationship, we have not included reference to NCAM1 in Table 2. This relationship needs further investigation before we can conclude its existence.

Collectively, we can conclude that *NLGN3* appears to be a CAM of interest in the pathology of ASD-related GI disease owing to its expression in both the GI system and CNS, on top of its status as an ASD candidate gene (16, 210). Autoantibodies against paranodal CAMs in inflammatory autoimmune disease prove the importance of neuronal CAMs in axon myelination, which is implied to be impaired in ASD brains *via* white matter dysregulation, but the origin of this dysregulation is unclear (146, 164). Seropositive CIDP cases illustrate how dysfunction of neuronal CAMs, including CNTNs and CNTNAPs, leads to a neuroinflammatory response in the CNS (164). NRXN3, CNTN2, and CNTN4 stand out as key CAMs that play a role in AD-derived inflammation within the CNS (190, 192, 204). Regulation of synaptogenesis by CAMs in the development of neural pathways may also have implications in both ASD and AD. A summary of neuronal CAMs that are associated with inflammatory diseases can be found in Table 2.

## Glial Cells and Neuronal Cell Adhesion Molecules

### Glia in Autism Spectrum Disorder

Glial cells encompass microglia, astrocytes, and oligodendrocytes, all found within the CNS (217). Collectively, glial cells have a major role in neuroinflammation and neurodegeneration, in addition to neuronal repair after insult (218, 219). Glial cells are sensitive to environmental cues within the CNS, such as inflammation or injury (220). Microglia act as resident macrophages of the CNS and once activated, produce proinflammatory cytokines and mediators, proliferate, migrate, and even present antigens to T cells (221). The secretion of proinflammatory cytokines, for instance, IL-1β and TNF-α, by microglia recruit immune cells to escalate the immune response and initiate the activation of astrocytes (39). Astrocytes have a similar role in inflammation as microglia. They are responsible for blood-brain barrier maintenance, immune cell activation, secretion of proinflammatory cytokines, and the induction of inflammatory-associated signaling cascades (222). After acute inflammation has been resolved through glial activation, microglia can regulate their own deactivation by the secretion of anti-inflammatory cytokines such as IL-10 and TGF-β (223). When the homeostasis of the CNS is altered, chronic activation of microglia or astrocytes can occur. This results in a prolonged inflammatory response, consequently causing damage to neuronal cells (224). Often, this is a characteristic of neurological or inflammatory disease (224). Oligodendrocytes myelinate axons within the CNS, which are important for synapse transmission and neuronal communication (225). As explained previously, dysfunction of myelination leaves neuronal cells unprotected against proinflammatory damage that may also impair cognition and sensory processing (144).

Examination of postmortem ASD brains identified microglial dysfunction as a feature of ASD pathophysiology (89, 226). Astrocytes undergo reactive gliosis and change morphology, much like microglia (227). A pivotal manuscript by Vargas *et al.* gave insight into the glial state within the brains of individuals with ASD. Dynamic neuroinflammation was observed within the cortex, cerebellum, and white matter of ASD subjects, as well as obvious activation of astrocytes and microglia (102). In another study, morphological changes were apparent in microglia from the prefrontal cortex of males with ASD, including decreased branching and thickening of the filopodia (226). Collectively, a neuroinflammatory state with involvement of glial cells, denoted by a change of phenotype, appear to characterize the pathophysiology of ASD. Glial reactivity could be, in part, a consequence of localized neuronal dysfunction onset by ASD and, therefore, could exacerbate synaptic and axonal aberrancy already present (226). Alternatively, glia may become activated in response to environmental cues such as LPS (89). In a rat model of LPS-induced MIA, microglia and astrocytes were activated within the fetal cortex soon after LPS administration (228). This poses an environmental source of glial activation resulting in a neuroinflammatory state. Regardless of the cause, prolonged glial activation is detrimental to neuronal health and consequently, cognitive function in ASD (224).

Microglia-derived cytokines, such as TNF-α, have been reported to regulate the pruning of neuronal synapses (229, 230). Efficient synaptic pruning is most active from the age of two and is vital for brain plasticity, which is thought to be lacking in ASD (231, 232). Researchers have found an increase in synapse number and evidence of under-pruning when examining the brains of children with ASD (233). This would agree with the onset of ASD-associated behaviors around the age of three, as well as behaviors in response to over-stimulation (3). However, there is also evidence that overproduction of TNF-α by microglia in ASD brains increases synaptic scaling, theoretically over-pruning synapses (79, 230). This may also have a detrimental effect on the formation of behavior pathways, especially during early neuronal development (234). As of yet, no consensus has been reached over these pruning hypotheses.

### Neural Cell Adhesion Molecules in Glial Cell Differentiation, Proliferation, and Phenotype

Glial cell proliferation is imperative in neural development. Astrocytes originate from radial glial cells and oligodendrocytes stem from oligodendrocyte precursor cells (OPCs), whereas microglia are thought to derive from resident macrophages in the yolk sac (225, 235). OPCs, astrocytes, and microglia are incredibly sensitive to sources of deterioration, including inflammation, and respond by proliferating (218, 225, 235). Dysregulation of cell proliferation and differentiation in the prefrontal cortex may be implicated in ASD (99).

It is important to acknowledge that various neuronal CAMs implicated in ASD are also expressed by glial cells. NCAM1, for example, is expressed on the surface of astrocytes in the brain and is vital in axonal regeneration after neuronal insult (236). However, there is further evidence that NCAM1 may be an important molecule for regulating neuroimmune signaling. Studies reveal that astrocyte-derived NCAM1 can alter NF-κB activity in both bulk rat brain tissue and cerebellar granule neurons (123, 237). NF-κB-mediated transcription in neurons and astrocytes was also increased with NCAM1 homophilic binding, whereby purified NCAM1 was added *in vitro* (123). This indicates that NCAM1 actively moderates NF-κB activity in astrocytes and neurons, hence, altering the levels of inflammatory cytokines produced and subsequently, the neuroimmune response. The Ig domains of NCAM1 are also reported to inhibit astrocyte proliferation *via* inhibition of MAPK signaling (237). As astrocytes are a major source of cytokines in the CNS, their reduced proliferation presents as a mechanism by which NCAM1 can regulate inflammatory responses in the CNS. Importantly, the Ig domain of NCAM1 does share homology with other members of the IgCAM superfamily (238). Therefore, there is also the possibility that other neuronal IgCAMs implicated in ASD could also exert similar effects on astrocytes, initiating NF-κB signaling or regulating astrocyte proliferation. The introduction of purified NCAM1 to rat forebrain astrocytes inhibited astrocyte proliferation, even with the addition of growth factors (239). Likewise, the application of anti-NCAM1 IgG showed the same inhibition of astrocyte proliferation (237, 239). Genetic variation in *NCAM1*, as demonstrated in ASD subjects by Zhang et al., may result in the production of a structurally ineffective form of NCAM1, meaning unrestricted astrocyte proliferation may occur in ASD brains in response to an inflammatory stimulus (240). In turn, this may contribute to the increased brain volume and astrocyte density observed in human ASD brains and ASD mouse models (99, 241). A polysialylated form of NCAM (PSA-NCAM) has also been implicated in postnatal spinal cord myelination in mice (242). PSA-NCAM, a post-translational modification of NCAM, is expressed on the surface of demyelinated axons, reactive astrocytes, and OPCs (216, 242). PSA-NCAM seems to be associated with OPC migration as well as myelination, as its expression is downregulated once the production of myelin begins (243). Alterations in myelination and white matter have been associated with ASD pathology, possibly linking these findings to PSA-NCAM (146, 244). Although, it is still unclear if PSA-NCAM may regulate the way OPCs respond to inflammatory stimuli or influence their production of inflammatory cytokines. Altogether, it appears that NCAM1 may regulate astrocyte proliferation in response to inflammatory stimuli and alter NF-κB signaling in both neurons and astrocytes. Additional research is still needed to better understand this process and to ascertain if other IgCAMs may perform similar roles in modulating neuroinflammation.

### Contactins and Contactin-Associated Proteins in Glial Cell Differentiation, Proliferation, and Phenotype

Another family of proteins that may alter neuroimmune responses is the CNTNs and their interacting partners, the CNTNAPs. CNTNAP1-deficient mice were found to have increased Notch signaling, which in turn promoted astrocytogenesis within the cerebral cortex (245). CNTNAP1 is expressed by the radial glial cells that differentiate into astrocytes, however, no changes in radial glial cell number were observed in CNTNAP1-deficient neonatal mice brains (245). Most radial glial cells transform into astrocytes shortly after birth, leaving differentiated astrocytes to generate new astrocytes (217, 246). During neuroinflammation, astrogliosis occurs, whereby Notch signaling promotes the proliferation of local astrocytes (247). Although CNTNAP1 deficiency may not directly alter the number of astrocytes or astrocytic function, it may alter Notch-mediated astrocyte proliferation (245). Similarly, CNTNAP2 does not appear to directly affect microglia or astrocyte number, however, it could influence astrocyte progenitor number. CNTNAP2-deficient mouse models revealed that the number of radial glial cells were decreased in the hippocampus of 5- to 6-month-old mice compared to wild-type mice (248). No differences in the number of mature astrocytes were indicated, suggesting CNTNAP2 may regulate astrocyte progenitor cell number but not the number of differentiated astrocytes. Additionally, CNTNAP2-deficient conditions alter the way astrocytes respond to stimuli. CNTNAP2-deficient mice exhibited a greater number of reactive astrocytes in the hippocampus following an induced seizure compared to wild-type mice (22). Combined, this data proposes that CNTNAP2 may moderate astrocyte activity at two neurodevelopmental stages; *via* astrocyte progenitor cell number at a younger age and also altering how differentiated astrocytes respond to stimuli later in life. Further experimentation on early postnatal transgenic mice would provide an understanding of alterations in the glial population as neurodevelopment progresses.

**FIGURE 2:**
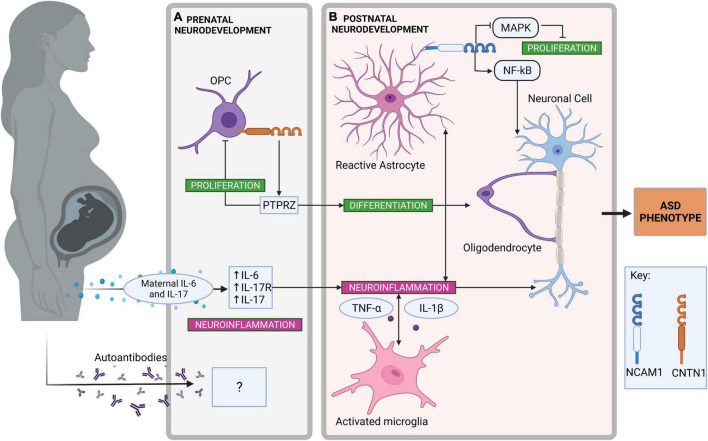
Neuroinflammatory pathways in prenatal and postnatal neurodevelopment contributing to autism spectrum disorder (ASD). **(A)** Neuroinflammatory pathways in prenatal neurodevelopment. Contactin-1 (CNTN1), expressed on the surface of oligodendrocyte precursor cells (OPC) promotes OPC differentiation to oligodendrocytes by interacting with receptor protein tyrosine phosphatase zeta (PTPRZ) (250, 258). Simultaneously, OPC proliferation is inhibited by the same PTPRZ interaction (250). The transfer of maternal proinflammatory cytokines such as interleukin (IL)-6 and IL-17 *via* maternal immune activation can promote neuroinflammation during prenatal neurodevelopment (115, 121). As a result, increased receptor IL-17 (IL-17R) expression is found within the fetal brain, enhancing further neuroinflammatory signaling (107). Although there is some evidence for autoimmune antibodies in association with ASD, their origin and effect on neuroinflammation in the developing ASD brain is unclear (140). **(B)** Neuroinflammatory pathways in postnatal neurodevelopment. Astrocytes are activated by proinflammatory cytokines and neuroinflammation in the central nervous system (227). Neural cell adhesion molecule-1 (NCAM1) expressed on the surface of astrocytes can activate nuclear factor-κB (NF-κB)-mediated transcription of proinflammatory genes (123). NCAM1 can also inhibit astrocyte proliferation through the inhibition of mitogen-activated protein kinase (MAPK) signaling (237). Changes to OPC proliferation have been observed in ASD brains, which can alter the production of myelin and possibly expose neuronal cells to inflammatory insult (146). Microglia are also activated by proinflammatory cytokines and neuroinflammation in the central nervous system (220). Activated microglia to produce proinflammatory cytokines including IL-1β and tumor necrosis factor (TNF)-α (39). Created with BioRender.com.

Altered OPC proliferation has been implicated in ASD pathogenesis alongside abnormalities of the white matter within the brains of those with ASD (146, 249). Analysis of differentially expressed genes in syndromic ASD models supports dysregulation of oligodendrocyte number (244). PTPRZ (receptor protein tyrosine phosphatase zeta), an interacting partner of CNTN1, is expressed in astrocytes, OPCs, and oligodendrocytes in the adult CNS (250). In the CNS, glial PTPRZ interaction with neuronal CNTN1 triggers cell signaling between glia and neuronal cells, promoting neuronal outgrowth important in neurodevelopment (251). CNTN1 is able to bind to PTPRZ at its Ig domain (252). A previous study observed that CNTN1-PTPRZ interaction on the surface of OPCs impairs OPC proliferation and induces oligodendrocyte differentiation (Figures 2A,B) (250). This indicates that CNTN1 and PTPRZ act as modulators of oligodendrogenesis. Although unexplored, alterations in CNTN1 (and other CNTN family members) expression may lead to changes in oligodendrocyte number or responses to inflammatory stimuli. Additionally, altered CNTN expression could contribute to dysfunctional myelination that exposes neurons to proinflammatory damage.

To summarize, glial cells are the key source of cytokines within the CNS and, therefore, are vital when considering the impact that neuroinflammation has in ASD (102). Neuronal CAMs seem to play a role, either directly or indirectly, in regulating glial activity. Importantly, neuronal CAMs may influence how glial cells respond to inflammation. One key example of this is CNTNAP2 acting as a potential moderator in astrocyte response after a stimulus (22). Over-proliferation of glial cells may contribute to ASD pathology, although there is some evidence of decreased OPC proliferation (99, 146, 226, 249). NCAM1 appears to directly regulate astrocyte proliferation *via* NF-κB signaling and may contribute to the increased brain volume observed in individuals with ASD (237, 239). CNTN1 may indirectly regulate OPC proliferation through the interaction of PTPRZ, which subsequently may affect neuronal myelination (250). These ideas are summarized in Figures 2A,B, depicting the role of neuronal CAMs in neuroinflammatory pathways during prenatal and postnatal neurodevelopment that may contribute to ASD. PSA-NCAM is expressed during early neurodevelopment making it a good marker to investigate the role of neuronal CAMs during behavioral pathway formation (216). Future investigations may be able to utilize PSA-NCAM to further explore the role of CAMs in inflammatory systems during neurodevelopment.

## Future Directions

It is believed that both genetic and environmental factors play a role in the pathology of ASD (16, 24). Inflammation has been identified as an environmental risk factor for ASD (220). There is strong evidence highlighting the presence of immune dysfunction in those with ASD, as well as the characterization of ASD-associated behaviors in MIA models (24, 36, 80, 109, 253).

In a healthy model of inflammation, immune cells proliferate and produce proinflammatory cytokines in response to pathogens or immunogenic materials (67). This response is facilitated by signaling pathways that can be activated by cytokines including the Notch, NF-κB, and MAPK signaling cascades (41, 55, 254). Chronic inflammatory signaling can occur if there is dysfunction of the immune system, resulting in unnecessary tissue and cellular damage, instigating the pathogenesis of autoimmune or inflammatory disease (41, 224).

Abnormally elevated proinflammatory cytokines (most commonly IL-1β, IL-6, IL-8, and IL-17) are consistently observed in the CSF and blood of children and adults with ASD (73, 74, 96). Although it is not fully understood, several studies have demonstrated that chronically elevated levels of these proinflammatory cytokines impair neurodevelopmental processes and neural cell function, including impaired synaptic pruning by microglia, irregular migration of neurons, and altered synaptic plasticity (57, 101, 230). It could be of particular interest for future studies to investigate whether specific cytokine profiles can be associated with inflammation originating from different environmental triggers (e.g., upregulated IL-6 and IL-17 are often associated with MIA) (Figure 2A) (110). Additionally, it is not yet certain which cell types, either in the CNS or the periphery, are the main drivers of inflammation in ASD (and the main cell types affected by the chronic inflammation).

Although it is unclear how the neuronal CAMs commonly implicated in ASD may mediate inflammation, investigating other disorders linked to ASD with an inflammatory or immune component can advise potential molecular mechanisms. As discussed in previous sections, the gut-brain axis provides the opportunity for peripheral inflammation to influence neuroimmune signaling and glial activation within the CNS through cytokines and vagal innervation (135). Most likely, GI inflammation is triggered by an exogenous source, such as in the case of food intolerance. In cases of ASD with GI dysfunction, the treatment of GI disorders to reduce inappropriate inflammation could improve the consequences of neuroinflammation. NLGN3 is of distinct interest concerning GI inflammation and ASD, owing to its interactions with ADAM proteins which may influence the production of proinflammatory cytokines in the gut and CNS (16, 210). Further epigenetic studies to explore environmental influences on *NLGN3* expression, and whether its dysregulation alters proinflammatory cytokine production, may be pertinent. There is also some evidence supporting a link to autoimmunity in dysregulation of the immune function in ASD, but literature is unclear on the origin of these autoantibodies (Figure 2A) (140, 148, 255). Additionally, there is no proof, as of yet, of the presence of autoantibodies against neuronal CAMs in those with ASD. Autoantibodies against paranodal CAMs in CIDP and MS demonstrate the importance of neuronal CAMs in the protection of neuronal cells against inflammatory damage, however, it remains uncertain how this is relevant to ASD (164). Although ASD is not typically characterized by neurodegeneration, an increase in secreted β-amyloid in the plasma of children with severe ASD identifies an association between the pathology of ASD and AD (151). CNTN4 regulates APP processing, whilst CNTN2 seems to have an association with Aβ (189, 190). The disruption of APP processing pathways may interfere with the balance of Aβ production, the clearance of which is mediated by activated astrocytes and microglia, causing a neuroinflammatory response (155). Therefore, altered expression of these CNTNs may increase Aβ production, contributing to neuroinflammation (153, 189, 192). Future research into the mechanisms by which CNTNs regulate APP processing, and whether dysregulation of this can alter glial cell activity, may reveal more about CNTNs functional role in modulating neuroinflammation.

Glial cells are the key source of cytokines within the CNS and, therefore, are vital when assessing neuroinflammation in ASD brains (102). In response to inflammation, glial cells change their morphology and proliferate (39). There is evidence that neuronal CAMs can play a direct or indirect role in the regulation of glial activity and, therefore, may influence glial responses to inflammation. CNTNAP2 may moderate astrocyte activity by influencing astrocyte progenitor cell numbers and affect how astrocytes respond to external stimuli (22, 248). Compellingly, NCAM1 has emerged as a key player in regulating neuroinflammatory cascades. NCAM1 homophilic binding can initiate NF-κB-mediated transcription in neurons and astrocytes, instigating a proinflammatory response (237). NCAM1 appears to directly regulate astrocyte proliferation (Figure 2B) and its dysregulation may, in part, account for the increased brain weight observed in individuals with ASD (237, 239). Additionally, CNTN1 may indirectly regulate OPC proliferation through interacting with PTPRZ (Figures 2A,B), which subsequently may affect neuronal myelination (250). Myelin is paramount in protecting axons from damage that may impair cognition and sensory processing (144). Further definition of neuronal CAMs regulatory role in glial activity and response to inflammation is missing from current literature. Experiments investigating CAM expression in glia at different developmental stages, and if neuroinflammation arising from this dysregulated expression can be treated in later life, would be of value for targeted anti-inflammatory therapeutics in ASD. Specifically, pre- and immediately postnatal neurodevelopment would be of interest, owing to ASD phenotypes presenting before the age of three (1).

The concept that neuronal CAMs may mediate or influence inflammatory cascades is largely unexplored in current literature. This review highlights the available evidence on the potential part neuronal CAMs play in neuroinflammation, with a particular focus on ASD. Further investigation into the role of neuronal CAMs within the context of inflammation is clearly warranted and would advance our understanding of neuroinflammation in ASD pathology.

## Author Contributions

AO-A: concept, research design, editing figures and artwork, and manuscript writing and editing. ME: research, generating figures and artwork, and manuscript writing. JG: research and manuscript writing and editing. LY: research and manuscript editing. All authors read and approved the final manuscript.

## Conflict of Interest

The authors declare that the research was conducted in the absence of any commercial or financial relationships that could be construed as a potential conflict of interest.

## Publisher’s Note

All claims expressed in this article are solely those of the authors and do not necessarily represent those of their affiliated organizations, or those of the publisher, the editors and the reviewers. Any product that may be evaluated in this article, or claim that may be made by its manufacturer, is not guaranteed or endorsed by the publisher.
